# Supplementary material for: Using big data to search for possible geographic clustering of Congenital Heart Disease (CHD) across Australia
Source: PLOS Digit Health. 2026 Feb 26;5(2):e0000916. doi: 10.1371/journal.pdig.0000916 (PMC12944802; doi:10.1371/journal.pdig.0000916)
Supplement: Attachment — Submitted filename: response_to_reviewers_20251125.pdf [file pdig.0000916.s001.pdf]

2 December 2025

Dear Reviewers,

Thank you for the thoughtful feedback on our submission “Using Big Data to search for possible Geographic Clustering of Congenital Heart Disease (CHD) across Australia”. We have gone through all your suggestions, addressing each point, in this revision. See below for our responses.

Regarding *data availability*, it has been noted that that the underlying data has not been made available. We agree that data availability is always preferrable, however, we will not be able to make these data available due to Australian privacy legislation that governs the use of this data. Access is only possible with prior approval from the Human Research Ethics Committee. The Data Availability Statement has been updated to provide more details here and provide contact avenues for anyone who does wish to obtain approval for data access.

Reviewer 1 Comments

First sentence of abstract has parentheses (cancer, neurological) after several disease. These examples add nothing to the meaning and ruins the flow of the sentence.

As suggested, the parentheses have been removed from this sentence (line 13)

Line 31 "People living with congenital heart disease have overgone many changes over recent decades." People have overgone many changes? Or do you mean treatment options for people with CHD have changed over time? I would suggest writing these clearer and with less editorialising.

As suggested, this sentence has been changed to “Outcomes for people living with congenital heart disease have improved greatly over recent decades.” Which is clearer and follows on to the next sentence that described the outcomes (“living longer”) and how the improvement has occurred (“surgical intervention has improved”).

You tested for a statistical association across all SA2 areas. I understand there are around 2,473, so setting a p-value of 0.05 means over a hundred or so will be statistically significance by chance alone without some kind of p-value correction. It's unclear if a Bonferroni (or other) correction was applied. If not applied, could you provide a rationale as to why it wasn't.

We note the suggestion concerning multiple comparison bias and have reviewed the best practice related to these statistical techniques. Our main finding (the study's pre-specified primary aim) is that the congenital heart disease population's geographic distribution is positively correlated with the general population. This is derived from the global coefficient, which is the result of a single test and therefore does not have potential for multiple comparison bias.

To provide less reliance on the p-value of the global coefficient, we have extracted two other important measures that support this conclusion. Firstly, the "Spatial Smoothing Scalar" provides a measure of autocorrelation for each variable in the comparison (i.e. autocorrection of the CHD population distribution and autocorrelation for Australian population distribution). Secondly, the Pearson's correlation of the neighbourhood weighted averages of the two analysis variables provides support to the global coefficient, regarding the overall positive correlation between the two observations. With these measures included, our conclusion is not supported by only one p-value. All analyses were still conducted in R, but a useful description is available in the ArcGIS documentation: https://pro.arcgis.com/en/pro-app/latest/tool-reference/spatial-statistics/how-bivariate-spatial-association-works.htm. These changes have been reflected in lines 126 to 136 and lines 162 to 166.

The next part of our analysis is exploratory; to measure local coefficients in each area, which (in aggregate) add up to the global observation, across Australia. We believe that this provides a useful way to describe local variations but as they are exploratory in nature, we believe that adjustment for multiple comparisons is not appropriate, in this case. This is now noted in the Methods (137-140) and, noting the point made by the Editor and the Reviewer, we have now added a more measured discussion of these results in the discussion (lines: 200-213).

Reviewer 2 Comments

The selected sample is large, but the registry used may introduce a selection bias excluding patients not followed in specialized services, or patients lost to follow-up.

We have noted this limitation of our data collection on lines 247-254. However, given that this is the first time a geographic analysis on the CHD population has been conducted on a population of this size, we believe that this study is still a robust and important contribution, despite the noted limitations.

The statistical analysis used allows for the definition of global and local spatial correlation, which answers the asked question, but other clustering methods using machine learning (k

means or DBscan) could have provided additional information independent of population density.

Machine learning methods (specifically k-means and DBscan) were explored in this research before applying the current bivariate approach. We felt that these machines learning clustering techniques did not provide helpful information that would identify important trends in the geographic distribution of the congenital heart disease population. Whilst they do identify clusters, The Australian population is very urbanised and it’s hard to know whether these algorithms just identify population centres as clusters. We felt our statistical technique needed to consider the distribution of the total population to provide true understanding of the characteristics of the geographic distribution of the CHD population. This point has been expanded upon in the discussion, lines 234 to 237.

Kind Regards,

Calum Nicholson, PhD Candidate.
Senior Data Analyst, ANZCHD Registry
Heart Research Institute, Sydney, Australia
